# Supplementary material for: Evolving Cognitive Dysfunction in Children with Neurologically Stable Opsoclonus–Myoclonus Syndrome
Source: Children (Basel). 2020 Aug 19;7(9):103. doi: 10.3390/children7090103 (PMC7552772; doi:10.3390/children7090103)
Supplement: Supplementary file 1 [file children-07-00103-s001.pdf]

Supplementary Materials:

Case 1

A 25-month old Caucasian boy was diagnosed with OMS after presenting with progressively worsening unsteadiness of gait over one to two months associated with new onset abnormal eye movements. Examination revealed opsoclonus, titubation, intention tremor and ataxia with intact cognition and age-appropriate development. His OMS severity score was 8/15. A neuroblastoma work-up was negative, including an abdominal ultrasound, brain MRI, MIBG scan and urine catecholamines. CSF analysis showed normal protein (0.14 g/L), glucose (3.0 mmol/L) and lactate (1.5 mmol/L) but was positive for oligoclonal bands. He was treated with high dose Prednisolone at 25 mg daily over a five-week taper, which led to improvements in symptoms and OMS severity score of 2/15. He had a good response to treatment initially, although his symptoms recurred following withdrawal of treatment. He was referred to our centre at the age of 3 years and 6 months at which point his OMS severity score was 6/15 and was re-commenced on high dose Prednisolone at 25 mg daily. As before, he responded well to treatment and was able to walk and run normally at his follow-up appointment six months later (OMS severity score 2/15). He experienced an exacerbation of his symptoms following an upper respiratory tract infection at the age of 4 years and 6 months. This was treated with high dose Prednisolone and IVIG (1 g/kg every four weeks), which led to resolution of his symptoms. He was continued on treatment with low dose Prednisolone and IVIG. Repeat MRI brain showed the presence of a small left thalamic infarct; MRA of the circle of Willis was normal. Cardiac echocardiogram was normal. There was no family history of stroke. There was no clinical evidence of connective tissue disorders such as Marfan's or Ehlers Danlos syndrome or of vasculopathy. Thrombophilia screen was negative. Anti-nuclear antibodies (ANA) and anti-neutrophil cytoplasmic antibodies (ANCA) screening were negative.

At the age of 5 years and 1 month, he developed acute onset severe headache, tremor, difficulty speaking and ataxia. MRI scan was performed, which showed an infarction of the left cerebellum and he was started on Aspirin and Dalteparin. He was not deemed suitable for further courses of IVIG due to the increased risk of cerebrovascular accident with this treatment. The following month, he was re-admitted because of worsening headache and generalized floppiness. A magnetic resonance angiogram (MRA) was carried out which showed dissection of the left vertebral artery. He improved following this admission and did not exhibit opsoclonus or nystagmus although he had significant truncal ataxia and intention tremor. A plan was made to convert him from Dalteparin to Warfarin and a repeat contrast angiogram was arranged at six months to determine the need for long-term anti-coagulation. This showed vertebral artery dissection on the contralateral side, which was asymptomatic. Following this, life-long anti-coagulation with Aspirin and Warfarin was commenced. During this period, he remained stable on low dose Prednisolone, with an OMS severity score of 2/15. He continued to be stable from symptoms related to OMS over the next few years with Prednisolone 5 mg on alternate days. A repeat MRI at the age of 7 years and 1 month showed a further subclinical cerebellar infarct. He underwent catheter cerebral angiography at the age of 8 years and 2 months, which did not reveal any abnormalities. Serial MRI and MRA between the ages of 9 and 13 years showed no acute abnormalities. He remained free of OMS symptoms and was weaned off Prednisolone at the age of 11 years and 6 months.

Prior to the onset of OMS, his early developmental history was unremarkable. In pre-school, at age 4 years 1 month, concerns were highlighted in a number of areas: the development of his speech and language skills, difficulties following classroom instructions, problems socialising with other children and displaying aggressive outbursts towards others when not under adult supervision. He was given a statement of special educational needs and remained in mainstream education with a high level of support. Formal neuropsychological assessment was performed at the age of 4 years and 11 months, during which his behaviour was at times challenging, but nonetheless his performance on the WPPSI-III was suggestive of 'average' intellectual functioning (VIQ 98; PIQ 101; General Language Quotient 94), but with significant problems with his attention, visuomotor skills,

behaviour and social interaction, which were considered to be impacting on his ability to engage in classroom learning.

He was reviewed at the age of 6 years 7 months, at which point he continued to be educated in a mainstream school with a statement of special educational needs and was reported to be making progress in all areas, albeit with a high level of adult support and careful management of his behaviour. The WPPSI-III was re-administered, but his short attention span and behaviour difficulties interfered with his ability to complete a number of subtests. His performance across a number of assessments was variable, with 'average' performance on tests of general language (GLC 91), but impairment on assessments of his general non-verbal skills (PIQ 67), visuomotor function and processing speed (PSI 60). He continued to display significant attention difficulties and impaired social development. Due to ongoing concerns about his academic progress, neuropsychological assessment was repeated at the age of 8 years 4 months. This confirmed an increased gap between him and his peers had developed, and that his intellectual skills, as assessed by the WISC-IV, now fell within a 'borderline' to 'exceptionally low' range (VCI 53, PRI 71, WMI 56, PSI 50) with reading skills as assessed by the WIAT-II falling in an 'exceptionally low' range (42). Further reviews at 9 years 5 months and 13 years old confirmed ongoing significant difficulties in his intellectual and academic abilities, with his performance across serial assessments being suggestive of a plateau in his cognitive development. Following completion of primary school, he transitioned to a special school for children with moderate learning disabilities.

Case 2

A Caucasian girl was diagnosed with OMS at 2 years 10 months after presenting with unsteady gait, vomiting and opsoclonus. Regressions in language and mobility were observed although these were regained within 6-9 months. She was admitted to hospital for three weeks and underwent brain computed tomography (CT), MRI and full body MRI. She underwent 12 rounds of Dexamethasone, speech and language, occupational therapy, and physiotherapy input. She gradually regained her pre-morbid skills and was discharged after 9 months. At 4 years, she was re-admitted to hospital following with a mild exacerbation of opsoclonus without myoclonus (OMS severity score of 3). Repeat blood tests, lumbar puncture and MRI were performed, which showed no abnormalities. She underwent a further 12 rounds of Dexamethasone – during this period energetic and challenging behaviour were observed but this subsided following completion of treatment. Post-treatment, she remained asymptomatic (OMS severity score of 0) for the entire duration of follow-up. Follow-up neuroimaging did not reveal any progressive or new changes.

All early motor and language milestones were achieved age-appropriately. She first presented with OMS symptoms at 2 years 6 months. She received speech and language, occupational therapy, and physiotherapy input during the first onset of symptoms and for approximately 6-9 months. Intervention was ceased as she regained all her pre-morbid skills. She was first seen for a neuropsychological assessment at 5 years 6 months, which found her general intellectual functioning to be within the 'average' range (FSIQ 100); her verbal and non-verbal reasoning were also in the 'average' range and her psychomotor processing speed and visual working memory fell in the 'low average' range. Wider neuropsychological assessments found appropriate skills in verbal episodic memory, sustained attention abilities and behaviour regulation. Qualitatively, she showed a good use and understanding of language which was consistent with the formal assessments carried out. Her visual motor integration abilities were found to be in the 'low average' range with a weakness in her fine motor coordination.

At 7 year 6 months a second neuropsychological assessment was carried out. This was due to some minor concerns around slow academic progress. Repeat imaging showed no abnormalities. Overall, there was a downward shift in her FSIQ, which fell in the low average range (81); VIQ, FRI, VSI, WMI and PSI were also found to be in the 'low average' range. Wider neuropsychological assessments found more pronounced difficulties in her ability to sustain attention, with a formal performance in the impaired range; her error pattern showed high numbers of omissions. Her attention difficulties were also noticeable through the assessment. No impairments were found in verbal and non-verbal episodic memory subtest. At school, she was on the inclusion register and

received in class support with her academic work. The school's biggest concern was with her ability to following instructions and complete academic tasks independently. Her attainment was below that of her peers who were of the same age, with a particular weakness in maths. However, she had made progress over the last academic year.

Case 3

A 17-month old girl was diagnosed with OMS after developing tremors in her legs and arms, which evolved into progressive ataxia over a period of two weeks and ultimately, loss of her ability to walk, sit up and talk (OMS severity score 11/15). She was admitted to hospital and full body MRI showed the presence of a paraspinal neuroblastoma at T4. She was treated with Dexamethasone and underwent surgical resection of the neuroblastoma after the first two rounds of treatment, leading to acute resolution of symptoms. However, she experienced a relapse at 20 months (OMS severity score 8/15) and was commenced on a three-weekly pulsed course of steroids instead of the initial four-weekly courses. At 21 months, she was unable to stand without support, but could sit with support. Her fine motor function including pincer grip for small objects was impaired, but she could play with large toys and finger-feed herself. Her opsoclonus was always rare and only when elicited by change of fixation. She was not able to hold her head consistently erect on trunk vertical, but able to reach and grasp objects with each hand and able to roll back to front. She was reviewed again at 22 months and found to be ataxic, with functional loss of her upper limb. Her quality of movement was jerky with frank cerebellar signs and head titubation (OMS severity score of 5). Treatment with Cyclophosphamide every four weeks was initiated till the age of 3 years 7 months. She was assessed by Speech and Language Therapy and Physiotherapy at approximately 2 years 6 months when her condition had stabilised and was noted to have recovered her previously lost skills and therefore, no input was required. Follow-up neuroimaging did not reveal any progressive or new changes. She remained asymptomatic since the end of treatment and for the duration of follow-up.

There were no concerns in early infancy, and she achieved all her early milestones on time. At 17 months, she showed her first signs of OMS. She was assessed by Speech and Language Therapy and Physiotherapy at approximately 2 years 6 months when her condition had stabilised and had recovered her previously lost skills; no concerns were noted, and no input was required. At both time points, there were no concerns in school. However, her mother had concerns regarding her ability to keep up with peers. At both assessments, she was noted to be very anxious and had difficulties separating from her mother. The first neuropsychological assessment was carried out at age 5 years 3 months. Her verbal and non-verbal intellectual ability were fell within the average to low average range, with an overall FSIQ was in the 'low average' range (88). Her performance on a test sensitive to processing speed was also in the 'average' range however she scored in the borderline range on a test of working memory. Wider neuropsychological assessments found no impairments in verbal episodic memory and visual-motor skills. Parent report and questionnaire responses were suggestive of some emotional difficulties and she was observed to be very anxious during the assessment. A repeat neuropsychological assessment was carried out at 7 years 8 months. At this point her intellectual ability fell in the intellectual disability range (FSIQ 68). VIQ, FRI, VSI and PSI were in line with this, while WMI was found to be in the 'low average' range. Wider neuropsychological assessments found impaired receptive language skills and sustained auditory attention, with no impairments in her expressive language skills. On tests of academic attainments, her reading and spelling were in the average range, but her maths was 'exceptionally low' (59). At this stage neuroimaging was repeated which was normal.
